# Early application of awake extracorporeal membrane oxygenation in pneumocystis jirovecii pneumonia complicated with severe acute respiratory distress syndrome: a case report

**DOI:** 10.3389/fmed.2023.1264928

**Published:** 2023-10-19

**Authors:** Qinglin Wu, Fulan Cen, Guowei Wang, Jia Huang

**Affiliations:** Department of Intensive Care Unit, Shenzhen Third People’s Hospital, Shenzhen, China

**Keywords:** extracorporeal membrane oxygenation (ECMO), venovenous ECMO (VV-ECMO), acute respiratory distress syndrome (ARDS), pneumocystis jirovecii pneumonia (PJP), case report

## Abstract

**Introduction:**

Patients suffering from severe acute respiratory distress syndrome (ARDS) are usually treated with mechanical ventilation. Extracorporeal membrane oxygenation (ECMO) has traditionally been considered a life-saving therapy and was reserved as a last resort when other treatment options were exhausted. However, this report outlines our successful initial experience with early implementation of awake venovenous extracorporeal membrane oxygenation (VV-ECMO) in a case of pneumocystis jirovecii pneumonia complicated by severe acute respiratory distress syndrome (ARDS), offering a promising new approach for recovery.

**Case presentation:**

We present a case report of the effective application of awake VV-ECMO in a 29 years-old man with severe ARDS caused by pneumocystis jirovecii pneumonia. The patient initially received antibiotic treatment and non-invasive ventilation (NIV) for respiratory distress, but these interventions failed to improve the worsening dyspnea that occurred in the patient. Following the combined antifungal therapy, high-flow nasal cannula (HFNC) oxygen therapy, and VV-ECMO for a duration of 7 days, the patient’s symptoms improved, showing relief.

**Conclusion:**

Awake VV-ECMO proved to be an effective treatment for critically ill patients with ARDS, avoiding the need for invasive mechanical ventilation. However, increased clinical evidence is needed to verify whether awake ECMO could be widely used in severe ARDS caused by other diseases or conditions.

## 1. Introduction

Pneumocystis jirovecii pneumonia (PJP) is a significant concern in patients who have received solid organ or hematopoietic stem cell transplants. These patients often require immunosuppressive medications to prevent rejection of the transplanted organ or graft-versus-host disease, which can weaken their immune system and make them more susceptible to opportunistic infections such as PJP (1). As a lung infection, PJP may result in respiratory failure, necessitating advanced respiratory support (2). Venovenous extracorporeal membrane oxygenation (VV-ECMO) has traditionally been employed as a salvage therapy to treat patients with respiratory failure under adequate sedation and analgesia (3, 4). In recent years, awake extracorporeal membrane oxygenation (ECMO) is often initiated earlier in the course of severe acute respiratory distress syndrome (ARDS) to avoid potential detrimental effects of mechanical ventilation, such as ventilator-induced lung injury (5). Awake ECMO, a novel therapeutic method allowing patients to remain conscious and breathe spontaneously without mechanical ventilation, is increasingly used due to its benefits such as lower incidence of ventilator-associated pneumonia, prevention of ventilator-induced diaphragm dysfunction, reduction of delirium, better rehabilitation, and treatment compliance (5, 6). However, early initiation of awake ECMO applied in a critical case of PJP with severe ARDS is exceedingly rare. Addressing this issue is urgent to improve the current status of severe ARDS cases, marked by stubbornly high mortality, prolonged length of stay (LOS), and increased hospitalization costs (7, 8). This report presents the successful early use of awake ECMO in PJP treatment, offering unique perspectives and insights for the management of these patients.

## 2. Case presentation

**Figure 1:**
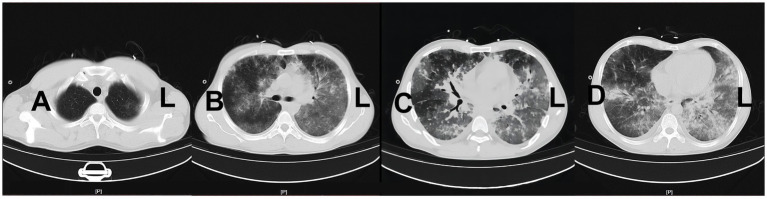
Reexamination of chest CT on the 15th day of admission before the application of ECMO.

**Figure 2:**
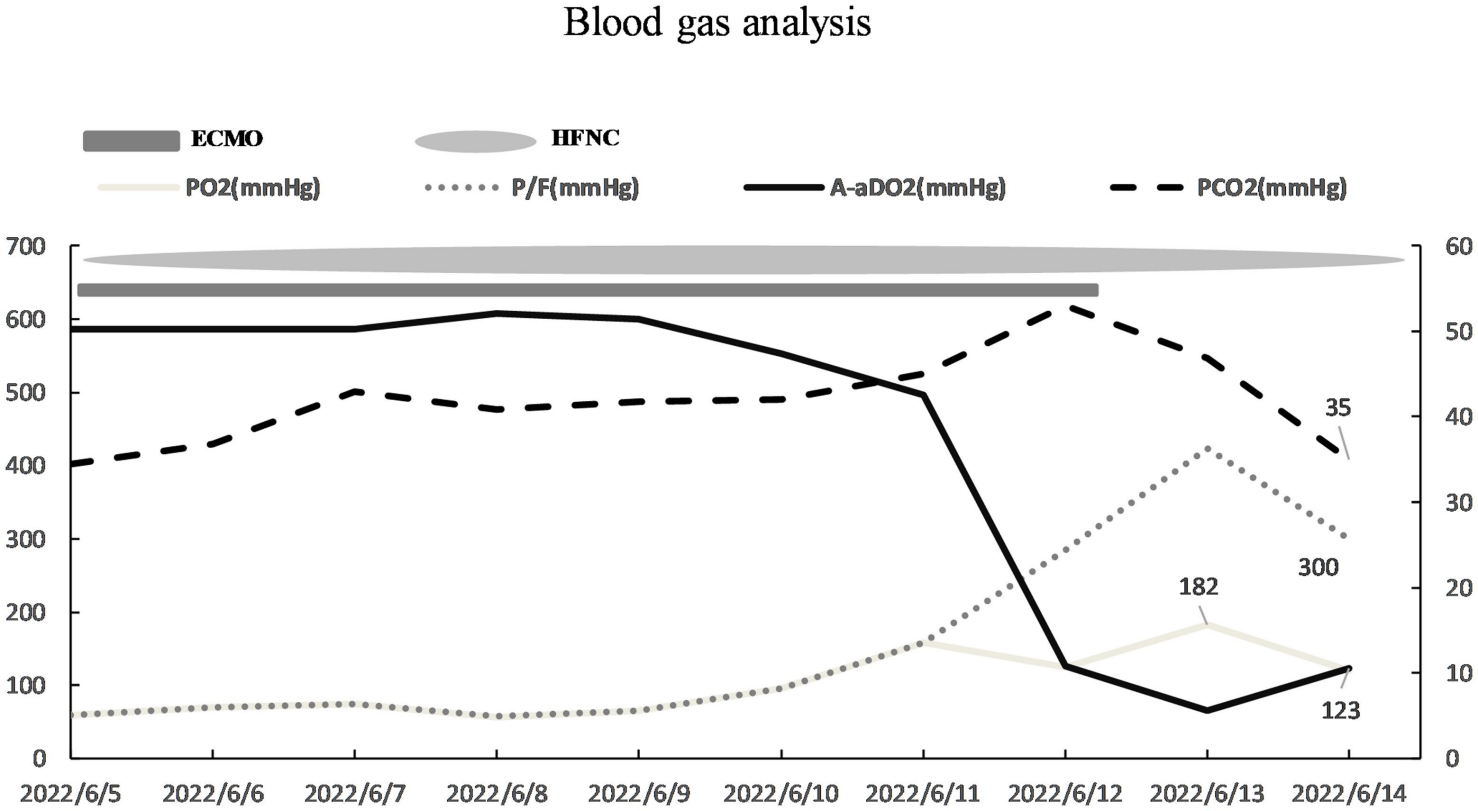
The result of blood gas analysis showed an improvement in the oxygenation index.

**Figure 3:**
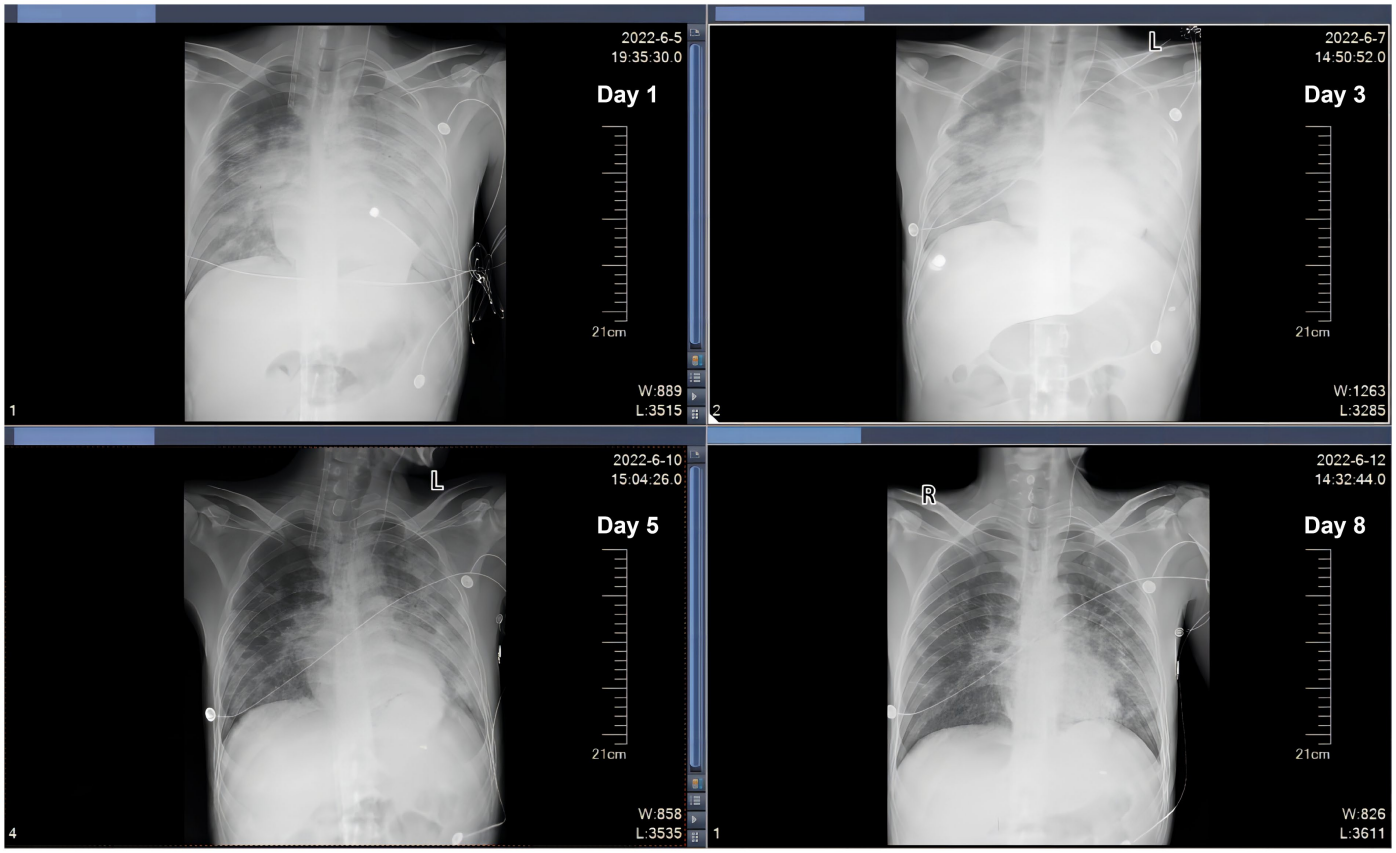
Imaging features of chest X-ray (D1, D3, D5, and D8).

**Figure 4:**
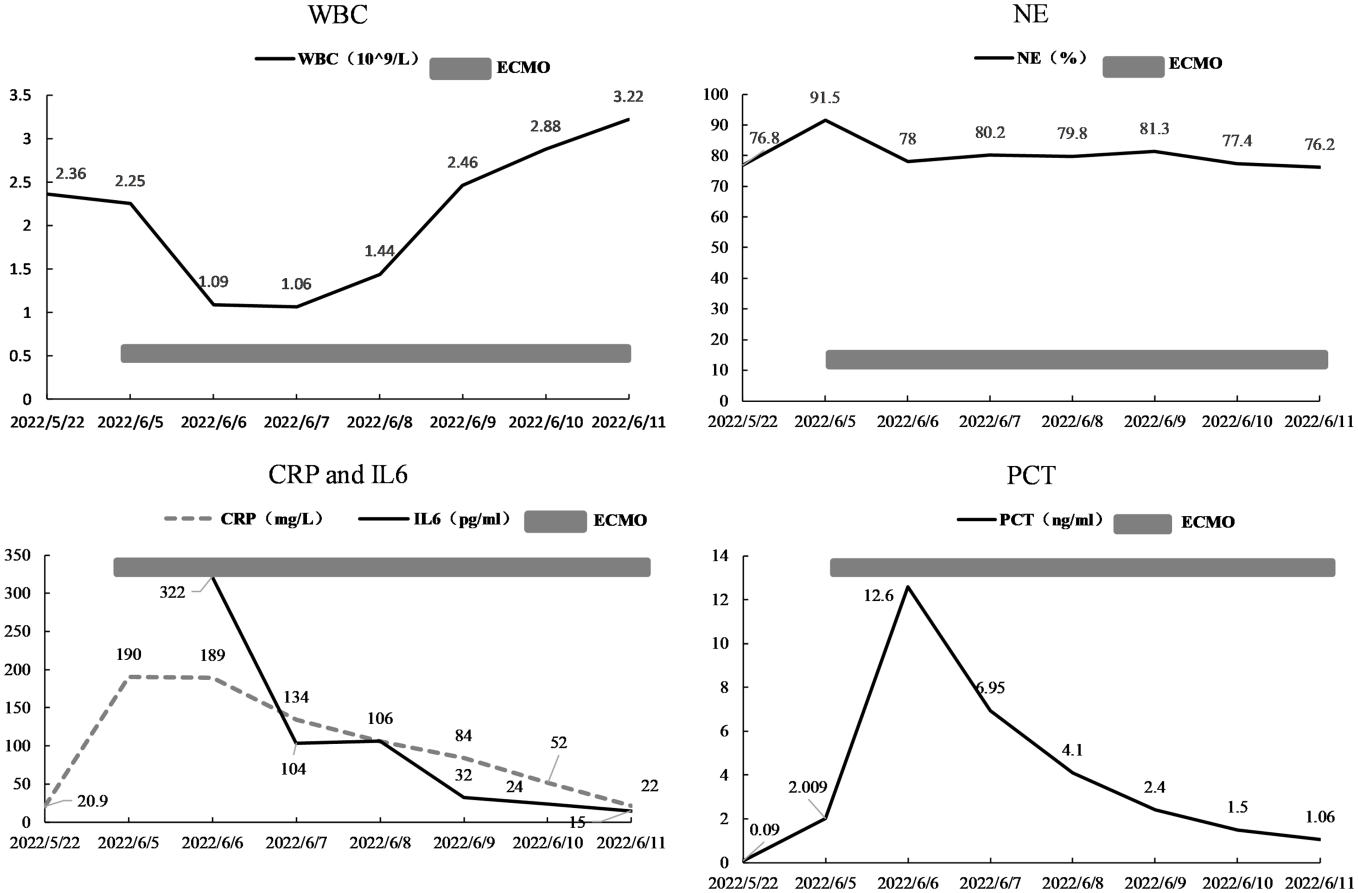
The change of WBC, NE%, CRP, IL6, and PCT.

A 29 years-old man was hospitalized in May 2023, presenting with a 3 days history of fever and dyspnea. The patient had previously been diagnosed with primary biliary cirrhosis (PBC) 9 years ago and had been on a long-term use of ursodeoxycholic acid (UDCA). He denied any related family history. On 6 November 2021, the patient was admitted to the hepatology department presenting symptoms of jaundice and lower limb edema. After admission, the patient was diagnosed with acute-on-chronic liver failure (ACLF) and underwent liver transplantation surgery after 1 year. The patient adhered to the prescribed regimen of tacrolimus and mycophenolate mofetil after liver transplantation to prevent transplant rejection. The physical examination of the patient revealed a temperature of 38.4°C, a heart rate of 84 beats/min, a respiratory rate of 20 breaths/min, an oxygen saturation (SpO_2_) of 99%, a blood pressure of 120/82 mmHg, and lung auscultation revealed diminished sounds with a few moist crackles heard. Other physical examinations were negative. Blood tests indicated a white blood cell count of 3.75 × 10^9^/L, a neutrophilic granulocyte percentage (NE%) of 77.6%, a procalcitonin (PCT) of 21.8 ng/mL, and a C-reactive protein (CRP) of 22 mg/L. The results of 1-3-β-D-glucan and G-lipopolysaccharide tests were negative, and the absolute counts of helper T lymphocytes and killer T lymphocytes were 220/μL and 261/μL. Upon admission, a chest computed tomography (CT) scan indicated minimal bilateral pulmonary exudates. According to the aforementioned findings, the preliminary diagnosis indicated that the patient had community-acquired pneumonia. Empiric therapy with cefuroxime was initiated upon admission for prophylactic purposes. Nevertheless, the patient’s pyrexia remained refractory, and there was an escalation of bilateral moist rales on the third day. Therefore, for further insights into the etiology, bronchoalveolar lavage fluid (BALF) samples were obtained through fiberoptic bronchoscopy, which revealed yellow foam-like exudates in the lungs that were positive for Gomori methenamine silver staining and displayed a small quantity of pneumocystis jirovecii encapsulation. Thus, the patient was diagnosed with PJP. The immunosuppressive agent and cefuroxime were discontinued, and caspofungin was introduced in conjunction with sulfamethoxazole (SMZ) for anti-infective purposes. Additionally, 40 mg of methylprednisolone was administered to mitigate the inflammatory response. After treatment, the patient continued to experience recurrent hyperpyrexia and dyspnea, accompanied by deterioration in vital signs. The patient’s temperature was recorded at 39.4°C, heart rate was 101 beats per minute, and respiratory rate was 32 breaths per minute. Then, despite the utilization of high-flow nasal cannula (HFNC) with an oxygenation flow rate of 50 L/min and FiO_2_ of 70%, as well as non-invasive positive pressure ventilation (NIPPV) with an IPAP of 18 cmH_2_O, an EPAP of 8 cmH_2_O, a frequency of 20 breaths/min, and FiO_2_ of 90% on alternate occasions, the pO_2_/FiO_2_ (P/F) ratio of arterial blood gas (ABG) was 100 mmHg. The P/F value satisfied the diagnostic criteria for severe ARDS. The patient experienced an exacerbation of dyspnea, and a reexamination of chest CT revealed an aggravation of lesions in both lungs (Figure 1). On the 15th day, the patient was admitted to the ICU under consideration of PJP and severe ARDS as diagnoses. He was still under hyperpyrexia and dyspnea physical examination and laboratory data showed a deterioration of general conditions: temperature: 39.3°C, heart rate: 102 beats/min, respiratory rate: 34 breaths/min, SpO_2_: 88% under high-flow nasal cannula oxygen (HFNC: oxygen flow rate of 40 L/min, FiO_2_ of 60%, and blood pressure: 115/74 mmHg). Arterial blood gas analysis showed severe hypoxemia: pH: 7.41, PaO_2_: 51.3 mmHg, PaCO_2_: 29.7 mmHg, and P/F 86 mmHg. The patient was expected to require mechanical ventilation for an extended period, which significantly increased the risk of mechanical complications. Meanwhile, given the patient’s desire for autonomy to manage his own behavior during dyspnea, without resorting to endotracheal intubation, coupled with the urgent need for oxygenation, the early employment of awake VV-ECMO represented the optimal course of action. With the patient’s consent, the VV-ECMO was connected to him. A 15F cannula inserted through the right internal jugular vein and a 23F catheter through the right femoral vein were placed for VV-ECMO under local anesthesia. ECMO parameters (blood flow velocity: 4.5 to 5.0 L/min, gas flow rate: 4 L/min, FiO_2_: 100%) were continued to be given with HFNC (Oxygen flow rate: 60 L/min, FiO_2_: 50%). On the basis of SMZ treatment, meropenem (1 g q8h) combined with vancomycin (1 g q12h) was given for antimicrobial therapy, dexmedetomidine (from 0.3 μg/kg/h to 0.5 μg/kg/h) was used for shallow sedation, and remifentanil (from 0.03 μg/kg/min to 0.05 μg/kg/min) for analgesia with the purpose to smooth the dyspnea. We maintained the RASS score from −1 to 0 of shallow sedation and controlled the spontaneous breathing rate at 8–15 breaths/min for lung protection. To prevent the formation of blood clots, we employed continuous infusion of heparin and frequently monitored the patient’s activated partial thromboplastin time (APTT) with the aim of maintaining a target APTT value between 60 and 80 s. Additionally, we administered enteral nutrition through a nasogastric tube to ensure a daily caloric intake of 2,100 kcal for the patient. On the 17th day, respiratory physiotherapy was initiated, which included daily use of the Portex Acapella Breathing Trainer was made at 6250 Shier Ring and sitting at the bedside with the intention of exercising respiratory muscles, preventing ICU-acquired weakness, and promoting pulmonary recruitment. On the 21st day, the manifestation of respiratory distress exhibited complete alleviation, the body temperature gradually regressed to the normal range, and the parameters obtained from blood gas analysis (Figure 2) as well as the radiological interpretation of the thoracic X-ray (Figure 3) depicted a gradual enhancement. According to Figure 4, the counts of NE, CRP, PCT, and IL-6 exhibited a gradual decrease over time, implying a potential amelioration or resolution of the infection. We partially adhered to the guidelines recommended by ELSO for weaning off VV-ECMO (9). We initiated a weaning trial at a PaO_2_ condition of 131 mmHg on supplemental O_2_ with an oxygenation flow rate of 40 L/min and FiO_2_ of 45% on HFNC while the fraction of delivered oxygen (FDO_2_) of VV-ECMO was 21%. Subsequently, we gradually reduced the sweep gas flow rate by 1 L/min from 5.0 L/min to our goal of 1 L/min over 4 h. Since the patient was able to tolerate the low sweep gas flow rate of VV-ECMO, we turned off the sweep gas for 4 h. Then, the arterial blood gas results obtained during the off-sweep gas trial demonstrated a PaO_2_ of 125 mmHg and pH of 7.41 without any excessive work of breathing based on the patient’s condition. Following an 8 h spontaneous breathing test mentioned above, the patient’s P/F was 277 mmHg, leading to the withdrawal of ECMO support. The patient was finally discharged from the ICU, and the LOS in ICU was only for 7 days without any adverse and unanticipated complications.

## 3. Discussion

In this case, the patient, with a history of liver transplantation surgery and administration of immunosuppressant, suffered from PJP leading to severe ARDS. Both the P/F ratio of ABG and chest CT imaging confirmed the diagnosis of severe ARDS. Conventional treatments for severe ARDS include tracheal intubation, protective ventilation, prone position ventilation, and deep sedation (10). ECMO was considered as the salvage treatment. It means that the patients have to endure at least 10–14 days of mechanical ventilation in conventional therapy, which may lead to a series of mechanical ventilation-related complications such as muscle atrophy under deep sedation, diaphragmatic dysfunction, difficulty in oral care of indwelling endotracheal tubes, and inevitable ventilator-associated pneumonia (VAP) (11). PJP can be secondary to immunocompromised patients in different stations, with a rapid disease progression and high mortality in non-HIV-infected patients (12, 13). However, the traditional mechanical ventilation treatment may induce series of complications as mentioned above. This suggests that new treatment methods and concepts need to be gradually innovated and practiced for better clinical outcomes.

In recent years, there have been several successful cases of early awake ECMO applied in different diseases, such as pulmonary hypertension, lung transplantation, broncho-cutaneous fistula, and COVID-19 (13–16). In a specific research study, patients supported with awake ECMO had higher survival rates than those primarily supported with intubated ECMO (10%, *p* = 0.011) (17). Based on the literature review and previous suggestions, awake VV-ECMO appears to be a more suitable option for individuals with isolated non-intubated lung injury, who are breathing spontaneously with severe ARDS. Thus, we innovatively applied early awake ECMO to a case of PJP complicated with severe ARDS and successfully completed the therapy due to effective management. First of all, the patient selected for the treatment was young, adherent, awake, and had few prior diseases, which reduced the complexity and risk of the treatment. At the same time, the patient’s conscious state and ability to breathe spontaneously facilitated active participation of the patient in the entire treatment process, nursing care, rehabilitation exercises, psycho-social aspects, and psychological counseling, which created favorable conditions for the prompt removal of ECMO upon recovery (6). On the contrary, patients with severe ARDS who underwent invasive ventilator-assisted ventilation may encounter challenges during extubation and reintubation. These challenges are associated with prolonged mechanical ventilation duration, a significantly high ICU mortality rate of 50%, increased incidence of ventilator-associated pneumonia, heightened use of sedatives, and elevated healthcare cost (18, 19). Second, effective lung rehabilitation exercise, aimed to improve the lung volume, ventilation, and blood flow, was an important means of lung recruitment. In this case, we performed pulmonary rehabilitation exercises with early respiratory training equipment while the patient was under mild sedation. We then evaluated the effect of lung recruitment maneuvers using bedside ultrasound, electrical impedance tomography (EIT), and other methods (20). The pulmonary function, blood gas analysis, and chest X-ray imaging were observed daily while adjusting the ECMO flow rate. This helped to further evaluate lung compliance and determine the intensity of pulmonary rehabilitation treatment. Third, we meticulously complied with the prescribed protocols and guidelines to ensure stringent prevention and control of nosocomial infections within the hospital setting. Then, the implementation of meticulously administered analgesia and light sedation proved to be of utmost importance in ensuring optimal patient comfort and safety throughout the treatment process.

By carefully managing these aspects, the primary goal of lung protection was successfully achieved by effectively reducing the frequency of spontaneous breathing and mitigating the potential adverse effects associated with excessive ventilation volume caused by driving pressure. Ultimately, the patient with PJP complicated with severe ARDS secondary to immunodeficiency after liver transplantation was treated with early awake ECMO successfully for only 7 days. Importantly, this remarkable outcome was achieved without any untoward complications, such as the development of ICU-acquired weakness, catheter-related bloodstream infection, or the onset of septic shock, further highlighting the efficacy and safety of this novel treatment modality.

Undoubtedly, certain limitations were observed in this particular case. The criteria for selecting the patient limit the ability to establish the safety and efficacy of awake ECMO in elderly individuals, those with multiple comorbidities, poor compliance, or impaired consciousness. Furthermore, the unsuccessful application of awake ECMO in a severe case of H7N9 avian influenza indicates that awake and non-invasive mechanical ventilation may not always be suitable for managing severe ARDS (21). Consequently, a substantial body of clinical evidence is required to delineate the specific parameters regarding the ideal candidates, optimal timing, and appropriate methodology for implementing awake ECMO (22).

## 4. Conclusion

In this case, we present the effective application of awake VV-ECMO in the treatment of critically ill PJP patients, avoiding invasive mechanical ventilation. However, with the limitation outlined above, increased clinical evidence is needed to verify whether awake ECMO could be widely used in severe ARDS caused by other diseases or conditions.

## Data availability statement

The original contributions presented in the study are included in the article/supplementary material, further inquiries can be directed to the corresponding author.

## Ethics statement

The studies involving humans were approved by the Ethics Committee of Shenzhen Third People’s Hospital. The studies were conducted in accordance with the local legislation and institutional requirements. The participants provided their written informed consent to participate in this study. Written informed consent was obtained from the individual(s) for the publication of any potentially identifiable images or data included in this article. Written informed consent was obtained from the patient for the publication of this case report.

## Author contributions

QW: Data curation, Formal analysis, Project administration, Resources, Supervision, Visualization, Writing – original draft. FC: Investigation, Supervision, Writing – review & editing. JH: Investigation, Project administration, Resources, Supervision, Visualization, Writing – review & editing. GW: Review & editing.
